# A Systematic Review and Meta-Analysis of the Effect of Short Birth Interval on Infant Mortality in Ethiopia

**DOI:** 10.1371/journal.pone.0126759

**Published:** 2015-05-22

**Authors:** Abel Fekadu Dadi

**Affiliations:** Institute of public health, college of medicine and health science, University of Gondar, Gondar, Ethiopia; University of Alabama at Birmingham, UNITED STATES

## Abstract

**Introduction:**

Even though Ethiopia has been celebrating the achievements of MDG 4, still one in every 17 Ethiopian children dies before their first birthday. This is the biggest of the African regional average. Short birth interval is inconsistently reported as a risk factor by limited and independent studies in Ethiopia. Therefore, the purpose of this meta-analysis was to determine the pooled effect size of the preceding birth interval length on infant mortality.

**Methods:**

Studies were accessed through the electronic web-based search mechanism from PUBMED, Advanced Google Scholar, WHO databases and journals: PLOS ONE, and BMC, using independent and combinations of key terms. Comprehensive meta-analysis version 2 was used to analyze the data. An I^2^ test was used to assess heterogeneity. Funnel plot and statistical significance by Egger’s test of the intercept was used to check publication bias. The final estimate was determined in the form of odds ratio by applying Duval and Tweedie’s trim and fill analysis in the Random-effects model.

**Results:**

872 studies were identified on the reviewed topic. During screening, forty-five studies were found to be relevant for data abstraction. However, only five studies fulfilled the inclusion criteria and were included in the analysis. In all of the studies included in the analysis, the preceding birth interval had a significant association with under-one mortality. The final pooled estimate in the form of the odds ratio for infant mortality with a preceding birth interval of less than 24 months was found to be 2.03 (95% CI: 1.52, 2.70, random effect (five studies, n=43,909), I^2^=70%, P<0.05).

**Conclusion:**

In Ethiopia, promoting the length of birth interval to at least two years lowered under-one mortality by 50% (95% CI: 35%, 63%).

## Introduction

Decreasing childhood mortality is the focus of the governments all over the world. The United Nations enshrined the right to life in the Declaration of Human Rights for children and this was reaffirmed in the Convention of the Rights of the Child[1]. Still millions of under-five children lost their life worldwide. According to the estimation of Population Reference Bureau 2012, on average, 41 children per 1000 live births were died globally before reaching their fifth birthday. Most infant deaths occur in the less developed world (45 infant deaths per 1000 live births in the less developed world compared with five infant deaths per 1000 in more developed countries) [2].

In Ethiopia, one in every 17 children dies before celebrating their first birthday, and one out of 11 children dies before their fifth birthday. The Ethiopian Demographic and Health Survey (EDHS) conducted during 2000, 2005 and 2011 showed significant reduction trend particularly for under-five mortality and partly for infant mortality but the case are different for neonatal and post-neonatal mortality. Infant mortality was registered a 39% and under five mortality rate was registered a 47% reduction [3–5].

The Millennium Development Goal (MDG) 4 had a target to reduce under-five mortality rate by two-third over the years 1990 to 2015. Even though current reports depicted the achievement of MDG 4 at the national level, the Ethiopian Ministry of Health has started to make plans for reducing under-five mortality rates to below 30 deaths per thousand live births by 2035 [6].

Approximately more than 42% of under-5 mortality in Ethiopia was attributable to infant and neonatal deaths [7]. Ethiopia has experienced a high infant and neonatal mortality compared to the average rate for the African region overall. Therefore, over the last decade, neonatal death has gained importance on the world policy agenda because the Millennium Development Goal (MDG) for child survival cannot met without substantial reduction in infant mortality [8].

In Ethiopia, the changes so far made on the area of infant mortality partly and neonatal and post neonatal mortality in particular were very stagnant.

The researcher hypothesis in this regard was related to the effect of short birth interval in increasing under one mortality. The current socio demographic transition in Ethiopia favors the increasing problem of short birth interval. Women spent a long time of their life at school and they are busy of leading their life, so they prefer to have a number of kids they desire within short period. This special area needs focus of policy makers so that the problem is not persistent to increase under-one mortality [4–5].

Different studies have shown that under-one mortality was influenced by multiple factors: maternal related factors, place of delivery, postnatal care service, and other neonatal factors play a significant role in reducing under-one mortality. Socioeconomic, demographic, health service delivery system, cultural practices, and technology are also important indirect determinants of under-one mortality [9–11].

Independent findings from different countries including Ethiopia have showed a relationship between a child’s chance of dying and specific fertility behaviors: preterm birth, short/long birth interval, and post term delivery. However, there are also inconsistent finding that shows the absence of association between these factors and child mortality [12–15].

There are highly possible and cost-effective interventions that could avert up to 72% of the neonatal death and can in terns leads to reduction of infant and under five mortality. This could only be achieved if countries adopt locally relevant and focused interventions guided by evidences [9].

Studies conducted so far in Ethiopia by taking specific fertility behavior as a risk factor for under-one mortality were few in numbers, independent, and reported inconsistent results. These independent and inconsistent study results can limit the opportunity to take targeted interventions to come up with a significant reduction in infant mortality.

Now a days using study result from meta-analysis can provide concrete evidence and have got due attention worldwide. So far, in Ethiopia, no meta-analysis was conducted to show the effect of preceding birth interval on infant mortality.

Therefore, the purpose of this meta-analysis was to determine the pooled effect size of preceding birth interval on infant mortality by reviewing a collection of evidences from limited studies conducted in Ethiopia.

## Methods

### Search approach and appraisal of studies

Studies for this meta-analysis were accessed through electronic web-based search. The researcher was used key terms: determinant, risk factor, cause, infant, neonatal, mortality, Ethiopia and combination of those words using the Boolean operator. The main databases searched were PUBMED, Advanced Google Scholar and WHO. The researcher was reviewed journals: PLoS one, BMC, The Ethiopian Journal of Biomedical Sciences, Ethiopian Journal of Health Science, and Ethiopian Journal of Health Development. After spotting relevant articles, their references were used to retrieve similar articles.

### Inclusion criteria

By thoroughly went through the content of the manuscript, those articles fulfilled the following criteria were included.

Publication year: Reports made from January 2000 onwards.

Language: Articles published in the English.

Publication condition: Publications published in peer-reviewed journals.

Study area: Only studies conducted in Ethiopia were included in the analysis.

Study design: Observational studies (case control, cohort and analytical cross sectional study) that were assessed the relationship between birth interval and under one year mortality.

### Exclusion criteria

Three blinded reviewers evaluated the abstracts as well as the full texts and performed the data extraction. After going through the full manuscript, abstracts that had methodological problems and rejected by the two independent reviewers were excluded.

### Data abstraction

This review was conducted from September 1, 2013—January 15, 2014. It was conducted in accordance with the Preferred Reporting Items for Systematic Review and Meta-Analyses (PRISMA 2009) statement with a 27- items checklist [16]. The relevance of the reviewed studies was checked based on their topic, objectives, and methodology. Preliminary assessment was made and some articles were excluded at the first step based on their topic. On the second step abstracts were seen and based on that, the articles were excluded if, they did not match to the current study objectives. For the rest, the whole content of the articles were accessed and selected based on the independent (length of preceding birth interval) and dependent variables (infant mortality) under review.

### Data analysis

The necessary information was extracted from each original study by using a format prepared in Microsoft Excel spreadsheet and transferred to Comprehensive Meta-analysis version 2 (CMA2) for further analysis. Heterogeneity was checked by using I^2^ test statistic [17]. As the test statistic showed significant heterogeneity among studies (I^2^ = 70%, p<0.05) the Random-effects model was used to estimate the DerSimonian and Laird's pooled effect. To identify the possible source of heterogeneity, Meta regression was undertaken by taking the sample size and different preceding birth intervals. The pooled effect was articulated in the form of odds ratio.

Funnel plot asymmetry and Egger’s test of the intercept in Random effects model was used to check publication bias [18]. As the results of the test suggested a possible existence of a significant publication bias (p>0.05 in Egger’s test), the final effect size was determined by applying Duval and Tweedie's Trim and Fill analysis in the Random-effects model.

## Results

### Explanation for original studies

The abstract search resulted in 872 references. Six hundred forty abstracts were found in PubMed and the remaining from different journals. After removing duplicated retrievals, 662 records remained, of which, 587 excluded during the initial assessment as their titles found to be irrelevant. For the remaining 75 records, their abstracts were accessed and screened. However, 30 excluded because they were not relevant in terms of the exposure and outcome variables. Therefore, 45 full text articles were accessed and assessed for eligibility based on the pre-set criteria. Finally, five studies were fulfilled the eligibility criteria and included in the analysis.

Out of the 45 full articles, 18 excluded because they were from other developed countries: India, China, Brazil, and America. Six of the remaining studies excluded because they were from other African countries: Nigeria [1], Ghana [1], Kenya [1], South Africa [2] and Uganda [1].

A nested case analysis from Butajira [19], a community based study from Gondar [20], a case control study from Gilgel Gibe Demographic Health Survey [21], and prospective case referent study from Butajira Demographic Health Survey [22] were excluded. Similarly, community based case control study from Jimma [23], a case control study from western Ethiopia [24], and a retrospective demographic data analysis from Agaro pastoralist community of Arsi, Southern Ethiopia[25] were excluded because their outcome measure was child mortality which was different from the outcome measure of this review (infant mortality).

A Cross-sectional study from Jimma was excluded because it did not met inclusion criteria for publication year [26]. Three studies: a study based on community and family survey in Northern Ethiopia[27], a case control study from hospitals in Addis Ababa [28], and a study from secondary data analysis of EDHS (2000&2005)[29] were excluded because of impossibility to extract data suit for this analysis.

A birth cohort study from Southwest Ethiopia [30], a study based on 2005 Ethiopian Demographic and Health Survey data [31], and a case control study from Region of Southern Nations and Nationalities [32] were excluded. Similarly, a cross sectional study from Region of Southern Nations and Nationalities [33], and a trend analysis from the three Ethiopian Demographic Health Survey data [34] were excluded because they lacked information on independent association of birth interval with the outcome.

**Fig 1:**
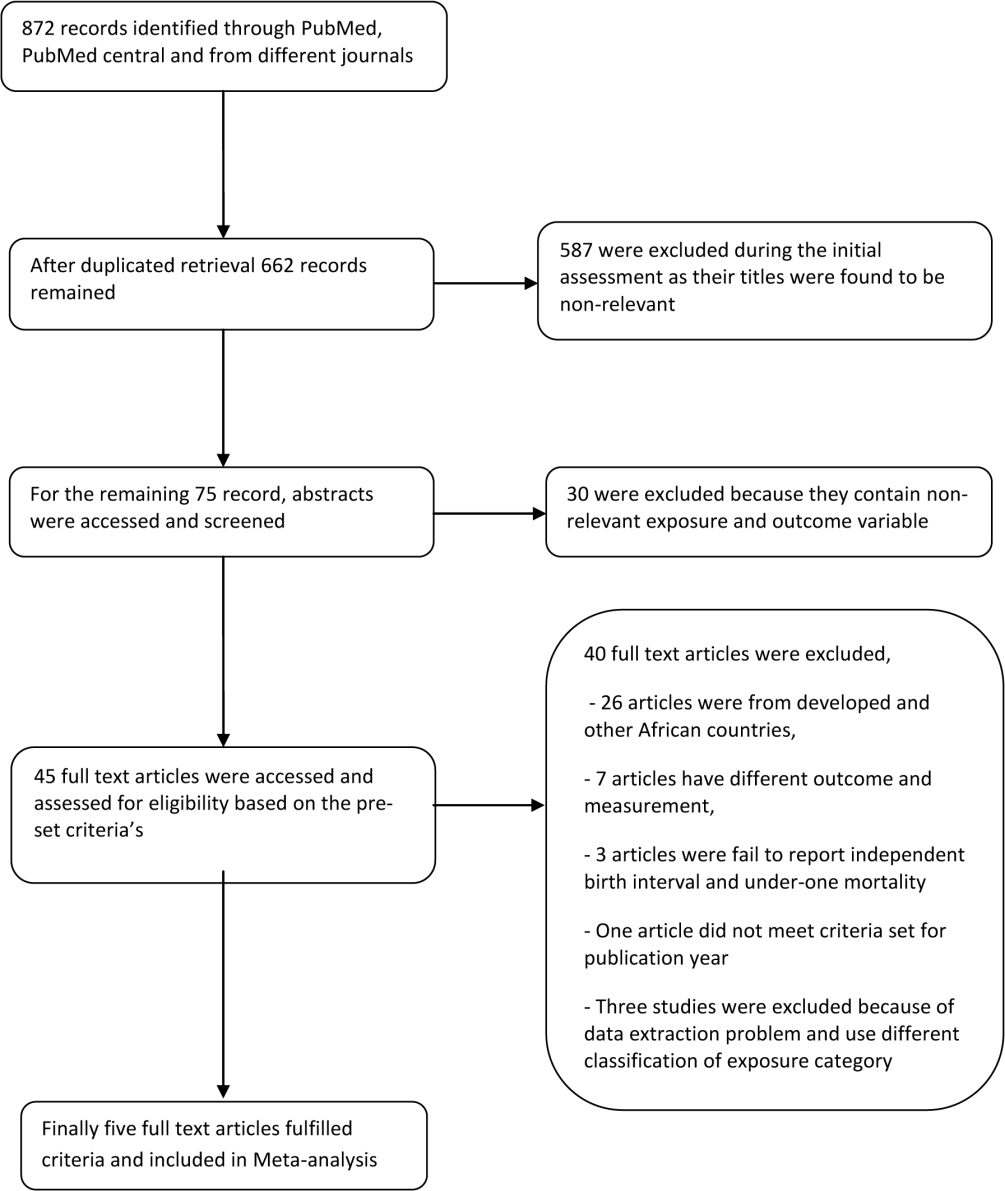
Flow diagram showing the procedure of selecting studies for meta-analysis, 2010–2013, Ethiopia.

Out of the five studies that were eligible and included in the meta-analysis, two studies were nation based studies [35, 36], and the other studies included were one from Northwest Ethiopia [37], one from south west Ethiopia [38], and the last was community based study from Kalu, Northeast Ethiopia [39] (Fig 1).

Regarding their study design: two were matched case control, two were extracts from the Ethiopian Demographic Health Survey, and the last article was a prospective longitudinal study. The original sample size for each study was ranged from 254 in the unmatched case control study from Gilgel Gibe to 32,428 for the National Ethiopian Demographic Health Survey based study. Regarding their quality: all of the studies were published on reputable journals and were re assessed by blinded reviewers prior to analysis and they were fit for their quality.

Concerning covariates adjusted in the studies: all potential covariates that could pose challenge to handle confounding variables were included except for limitation of secondary data and for few studies some variables like essential newborn practice, environmental factors were a challenges to got information.

In all of the five studies included in the meta-analysis, 43,909 live births were involved. Of which, 7,584 births had less than 24 months of preceding birth interval, while the remaining 36,325 births had an interval of greater than 24 months (Table 1).

**Table 1:** List of studies included to show the effect of length of preceding birth interval on under-one mortality, 2010–2013, Ethiopia.

S.N.	Author/year of publication	Study area	Study design	Total sample size	Under one children with Preceding birth interval < 24 months	Under one children with Preceding birth interval > = 24 months	Weakness of the study
1	Andargie G. 2013	Dabat DHS	Prospective longitudinal study	1,752	523	1,229	Small sample size of death Birth weight and other clinical conditions not measured
2	David P. 2010	Ethiopia	EDHS based	9,173	1,303	7,870	Problem of missing information for handling residual confounding factors
3	Dube L. 2013	Gilgel Gibe	Matched case control	254	39	215	Relatively small sample size
4	Yigzaw M.2010	Kalu,	Matched case control	302	43	259	Relatively small sample size
5	Mekonnen Y. 2013	Ethiopia	DHS based	32,428	5,676	26,752	Underreporting of deaths by mothers Lack of information on essential new born care practices
			Total	**43,909**	**7,584**	**36,325**	

### Pooled effect size

In random effect model, weight for every study was given based on individual study effect size and sample size [40]. In this case, the weight given for Andargie G. et al, David P. et al, Dube L.et al, Yigzaw M.et al and Mekonnen Y.et al. was 18.33%, 29.43%, 9.2%, 7.41% and 35.76%, respectively.

**Fig 2:**
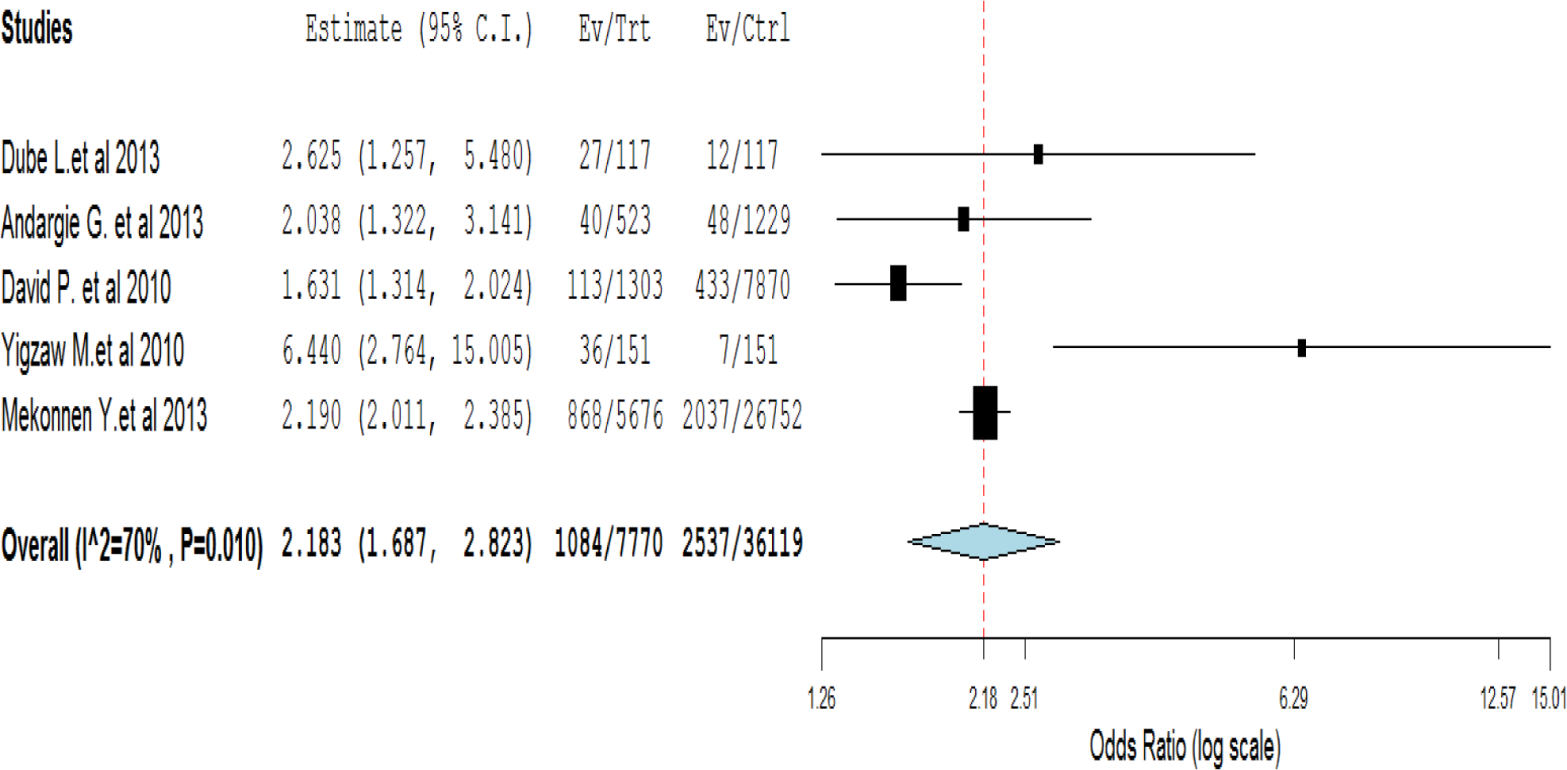
Shows forest plot of five studies to show the effect of preceding birth interval on under-one mortality, 2010–2013, Ethiopia.

In this model, all of the five studies included in the analysis were showed a statistically significant association between infant mortality and length of preceding birth interval. The pooled effect size of infant mortality among index infants with the length of preceding birth interval of less than 24 months in the form of odds ratio (OR) was 2.456 (95%CI: 2.245, 2.686) as compared to preceding birth interval greater than or equal to 24 months in the fixed effect model. However, the I^2^ test for heterogeneity showed significant difference among studies (I^2^ = 70%, P<0.05). So, the DerSimonian and Laird random effect model was used to determine the pooled effect size [41, 42]. Finally, the pooled effect size for infants with preceding birth interval of less than 24 months in the random-effect model became 2.18 (95% CI: 1.69, 2.82) as compared to those greater than or equal to 24 months (Fig 2).

According to the Meta regression analysis in the random effect model, sample size and effect size showed significant difference, i.e., the larger the sample size, the larger the effect size would be. Similarly, as preceding birth interval decrease the odds of under-one death increases.

Funnel plot of precision asymmetry as well as Egger’s test of the intercept was used to check publication bias [43]. On visual examination, the funnel plot was found to be asymmetric and Egger’s test of the intercept (B_0_) was found to be 0.513 (95%CI: 3.77, 4.79 P>0.05). This indicate the presence of publication bias which forces to conduct and report Duval and Tweedie’s trim and fill analysis to adjust the final pooled effect size.

**Fig 3:**
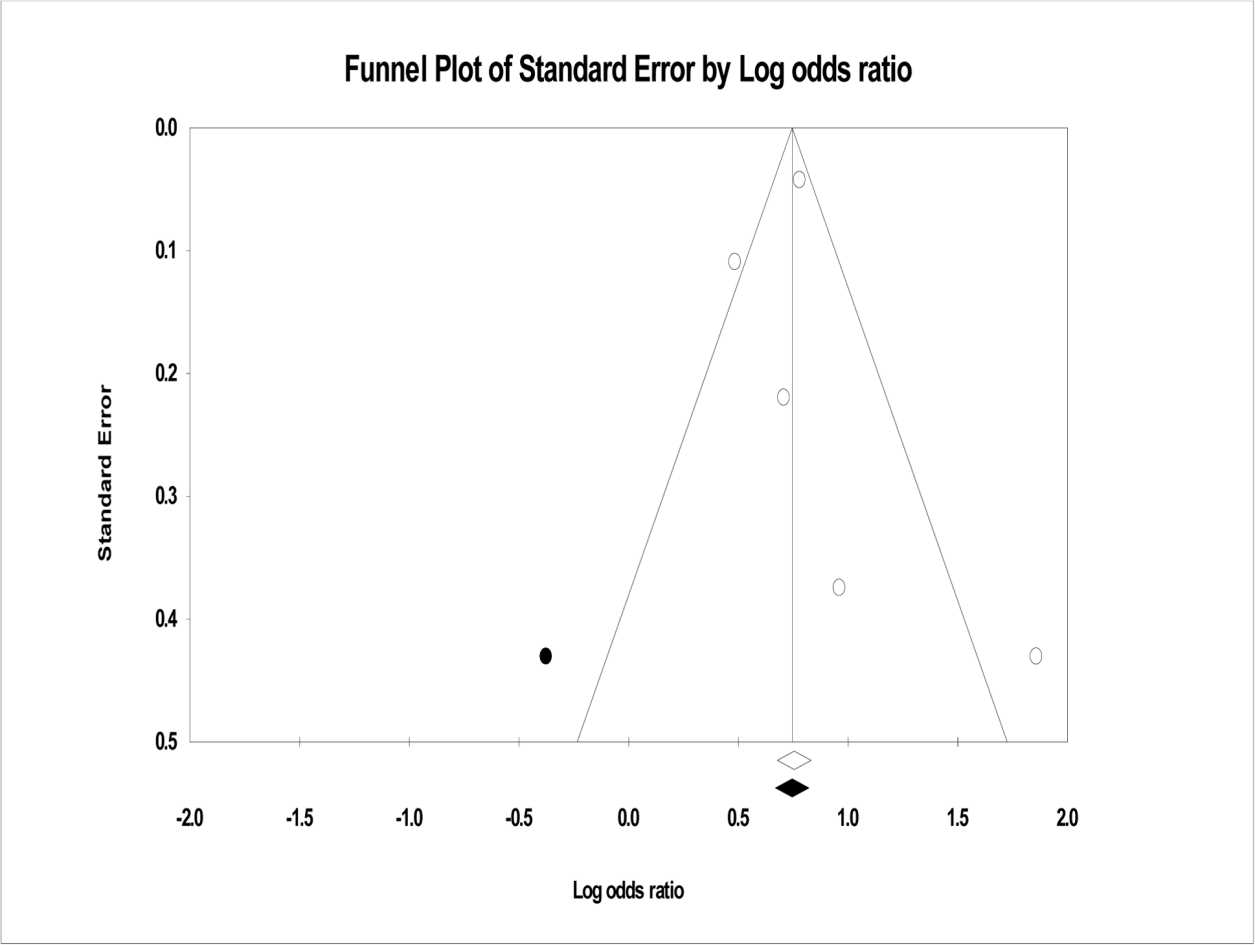
Shows funnel plot of five studies to show the effect of preceding birth interval on under-one mortality, 2010–2013, Ethiopia.

The program is looking for missing studies on the left side of the mean size based on random effect model. By using this parameter, the method suggests that one study was missing. So the final pooled effect size after trim and fill analysis in random effect model was found to be 2.03 (95%CI: 1.52, 2.70). This showed the presence of a significant association between the length of the preceding birth interval and infant mortality (Fig 3).

## Discussion

This meta-analysis attempted to assess the pooled estimate of the length of the preceding birth interval on under-one mortality in the context of Ethiopia.

The finding revealed the presence of strong association between the lengths of preceding birth interval and infant mortality. The length of preceding birth interval of less than 24 months was associated with a 50% (95% CI: 35% to 61.7%) augmentation of infant mortality in Ethiopia. As all of the included studies were from different parts of Ethiopia, it could best apply for the country to undertake different policy actions. Similarly, a Meta regression fitted to identify the source of variation in the estimate showed that a study with a large sample size was tended to increase the magnitude of the estimate, and very short birth interval was tended to associate with higher risk of infant mortality significantly.

“The adverse consequences of a short birth interval on infant and child survival have been attributed to the biological effects related to the “maternal depletion syndrome” where the woman might not fully recuperate from one pregnancy before supporting the next one may lead, for example, to anemia and premature rupture of membranes. (1) Behavioral factors associated with competition between siblings, such as competition for parental time or material resources among closely spaced siblings, (2) inability to give a child adequate attention if his or her birth comes sooner than desired time; and (3) disease transmission among closely spaced siblings” were among the published consequence of short birth interval [44].

Although studies from meta-analysis so far conducted on the area on specific research questions are not available, survey like EDHS report in different years, supported the finding of this study. Experience from developed countries also supports the current finding [9–13, 45– 50].

Because of lack of randomized controlled trial (RCT) on the topic, all available observational studies that were directly fit the research question and fulfilled the eligibility criteria were incorporated. Many authors proved that observational studies could give valid finding with moderate estimate when RCTs were not available to provide strong evidence. Therefore, the result of this study could provide a valid estimate in showing moderate evidence on the area [51–54].

A study from Addis Ababa indicated that maternal education was positively associated with shorter length of preceding birth interval. Educated women lost time going to school, so they need to get the desired numbers of children within left short period of time [55]. This was supported by the three EDHS report, which depicted the absence of change in median time of birth interval.

According to the 2011 EDHS report, in Ethiopia 77% of currently married women experienced risky fertility behavior of different magnitudes and 80% of the married women had the potential to give birth to children with elevated risks of mortality, and it was very devastating for under-one year old group [5]. One of the most risky fertilities was a short preceding birth interval (< 24 months). This showed the magnitude of the problem to pose the highest burden in the achievement of MDG and beyond set by the Ethiopian Government. Therefore, without tackling this practice it will be difficult to think about the attainment of the Goal set by 2035.

The use of the random effect model might overestimate the true magnitude and similarly using trim and fill analysis to avoid publication bias might also underestimate the true magnitude, so, it is vital to note this while interpreting and using this finding.

This meta-analysis may have some limitations as it is limited to a few publications written in the English and observational studies. Additionally, because of the nature of meta-analysis, that uses aggregated group data, other confounding factors that can affect infant mortality were not proscribed. This might have affected the estimate. Therefore, the findings of this meta-analysis would be best if interpreted in the context of both inherent limitations of the original studies and the current analysis. Tura G et al. base some of the wordings in this article on the article under the creative common license [55].

## Conclusion

This meta-analysis found that, in Ethiopia, promoting the length of birth interval to at least two years was associated with lowering under-one mortality by 50%.
